# The Effect of Sodium Chloride on the Physicochemical and Textural Properties and Flavor Characteristics of Sous Vide Cooked Duck Meat

**DOI:** 10.3390/foods12183452

**Published:** 2023-09-15

**Authors:** Man Zhang, Cuncun Fu, Mengfei Chen, Changhai Jin

**Affiliations:** College of Food Science and Engineering, Yangzhou University, Yangzhou 225127, China; mzhang100@yzu.edu.cn (M.Z.); f15518823513@163.com (C.F.); cmf15737113507@163.com (M.C.)

**Keywords:** duck meat, sous vide cooking, salt brining process optimization, flavor characteristics

## Abstract

This study was conducted to evaluate the effect of salt brining process parameters (salt concentration 0–15%, brining time 4–12 h, brining temperature 4–20 °C) on the quality of sous vide cooked duck meat by a single factor combined with response surface methodology (RSM). The sensory evaluation, physicochemical indexes (color, weight loss, NaCl content, TBARS value, and texture properties), and flavor characteristics were analyzed. The sensory overall mean score was applied as the evaluation index to optimize the brining conditions by RSM, and the optimum results contained a salt concentration of 11.69%, a brining temperature of 7.35 °C, and a brining time of 8.03 h. Under these conditions, the sensory overall mean score of duck meat was 8.59, with a relatively higher a* value and moderate NaCl content. GC-MS and odor activity value (OAV) results indicated that salt brining treatment significantly promoted the formation of the major odorants in duck meat, including pentanal, heptanal, octanal, (E)-2-nonenal, cis-4-decenal, decanal, 2,4-decadienal, (E,E)-2,4-decadienal, 1-heptanol, and 2-methyl-3-octanone, but decreased the content of hexanal, (E)-2-octenal, nonanal, (E,E)-2,4-nonadienal, 1-octen-3-ol, and 1-octen-3-one. 5′-nucleotides in duck meat were significantly increased after brining treatment. Therefore, salt brining treatment could be regarded as an efficient way to improve the sensory, aroma, and taste quality of sous vide cooked meat.

## 1. Introduction

Duck meat not only contains a large amount of unsaturated fatty acids but also is rich in trace elements, vitamins, and other valuable substances. In addition, duck meat products are an important source of high-quality protein for the human body [1,2]. Sous vide (SV) is a cooking method that places food raw materials in vacuum-sealed and heat-resistant food-grade plastic bags for processing at strictly controlled temperatures [3,4]. The processing temperature is usually lower than 100 °C, and the central temperature of the products is generally controlled at 60~80 °C [5]. Compared with traditional cooking methods, sous vide cooking has many advantages. Raw materials are cooked in vacuum-sealed packages and heated with stable heat migration, which can retain the original flavor by inhibiting the flavor substances caused by excessive lipid oxidation and preventing the volatilization of compounds in the cooking process [6], reduce the growth of aerobic bacteria and extend the shelf life of food by eliminating the risk of recontamination during storage [7]. Gok et al. reported that beef *m. gluteus medius* cooked by sous vide showed lower cooking loss and higher tenderness than those by an oven [8]. Rasinska et al. found that rabbit meat cooked by sous vide had lower cooking loss and higher content of lipids and sulfur-containing compounds compared with boiling and roasting [9]. Recently, studies about duck meat mainly focus on the properties of raw materials and duck meat products for sale (such as Nanjing salted duck) [10,11]. There are few studies on the effect of sous vide processing treatment on the quality of duck meat.

As a significant food ingredient, sodium chloride is usually used in meat products and can contribute to preserving products and extending the shelf life, enhancing flavor characteristics, increasing microbial stability, and improving color appearance [12,13,14]. Baskentli et al. reported that salt had a significant flavor enhancement effect on the model samples of beef soup and could improve the meat aroma and saltiness of the samples [15]. Moreover, salt improves the water-holding capacity of meat [16]. Salt can promote the dissolution of myofibrillar protein and activate protein hydration, thereby affecting the tenderness, cooking loss, and juiciness of meat products [17]. Du et al. found that the hardness of duck meat increased with an increase in salt concentration during the wet curing process [18]. However, excessive salt intake can cause various health issues, such as cardiovascular diseases and hypertension [19]. Health organizations around the world have called for reducing the average sodium intake of the general population by reducing the amount of sodium chloride in processed foods [20]. A reduction in salt content can lead to the creation of a variety of flavor substances by promoting the decomposition of protein oxidation and free amino acids in sour meat [21]. Many studies reported that salt concentration had a critical effect on the quality characteristic of processed meat products [22]. Yang et al. studied the effect of salt concentration (0%~10%) on the quality characteristics of pork jerky. Sensory results indicated that the color, juiciness, aroma, and tenderness of the samples were enhanced after pre-soaking in a 2% salt solution [16]. Xing et al. analyzed the volatile and non-volatile components of dry-cured pork samples with different salt contents (0%, 1%, 3%, 5%, 7%). The results showed that samples with a salt content of 3% had the highest content of free amino acids, and samples with 3% and 5% salt content had the highest peak area of flavor substances and taste index [23]. Other salt brining parameters, such as brining temperature or time, also affected the properties of meat products. Dimakopoulou-Papazoglou et al. demonstrated that an increase in curing temperature could reduce the viscosity of the permeating medium and improve the viscoelasticity of meat [24]. Thus, it could be seen that the addition of salt had an important effect on the quality of meat. In this study, a salt brining treatment was used on duck meat prior to sous vide cooking. It was reported that sous vide cooking combined with a certain salt addition could increase the moisture content and reduce the cooking loss of chicken breast ham compared to traditional cooking methods [25]. However, little information was known about the effect of salt addition on the quality properties of sous vide cooked duck meat.

Gas chromatography coupled with mass spectrometry (GC-MS) is the most widely used technique for the analysis of volatile compounds in food. Although a large number of volatiles can be identified by GC-MS, only the odor-active compounds contribute to the overall aroma of food products. The contribution of the flavor component depends not only on the concentration but also on its threshold value. The threshold is the minimum concentration for a volatile substance to be perceived [26]. It is generally reported that substances with an odor activity value (OAV) greater than 1 were the key odorants that contribute to the overall flavor [27], and the greater the OAV value, the greater the contribution to the sample. Sun et al. confirmed that eugenol, linalool, hexanal, anethole, diallyl disulfide, 2-octyl acetate, a-copanen, and 1-octen-3-ol were the key odorants in the storage of cooked beef meatballs by flavor dilution factor and OAV values [28]. Qi et al. detected 57 volatiles by gas chromatography–olfactometry–mass spectrometry (GC-O-MS) and identified 22 odor-active compounds with OAVs more than 1 as key aroma flavor substances in mutton soup [29]. Jiang et al. indicated that 19 key aroma active substances (OAV ≥ 1) had a great influence on salt-roasted chicken legs using GC-MS and OAV analysis, including (E,E)-2,4-heptadienal, ethyl cinnamate,1-octen-3-ol, furfural, octyl aldehyde, trans-2-nonenal, nonyl aldehyde, (E,E)-2,4-nonadienal, etc. [30]. Therefore, GC-MS combined with OAVs could be a useful method to explain the flavor composition and differences in sous vide cooked duck meat influenced by salt brining treatment.

In our study, a single-factor analysis combined with response surface methodology was used to evaluate the effects of brining conditions (salt concentration, brining time, brining temperature) on the eating quality of sous vide cooked duck meat. The flavor characteristics of salt-cured duck meat were analyzed by sensory analysis, GC-MS, and OAV value. The physicochemical indicators (color, weight loss, NaCl content, TBARS value) and textural properties were detected. There were previous studies about the optimal brining conditions of meat products. Duck breasts brined at a 15% NaCl solution had higher process yields, diffusion coefficients, and lower health risks [18]. Prior to drying, pork jerky samples pre-soaked in a 2% salt solution had the highest scores of overall acceptability [16]. The optimum salt curing conditions of sous vide cooked ducks in our study provide theoretical guidance for the preparation and processing of salted duck products.

## 2. Materials and Methods

### 2.1. Materials and Chemicals

Duck legs (235 ± 11 g, with bone and skin) from the same manufacturer were procured from RT-Mart (Yangzhou, China) and immediately frozen at −20 °C. Refined salt and Chinese rice wine were purchased at the Yangzhou RT-Mart supermarket (Yangzhou, China). Standard chemicals of chromatography grade were purchased from Sigma Aldrich Ltd. (St. Louis, MO, USA). The analytical grade chemicals were purchased from Sinopharm Chemical Reagent Co., Ltd. (Shanghai, China).

### 2.2. Sample Preparation

Duck legs (with bone and skin) with normal color and no surface damage were thawed at 4 °C, rinsed with water, and stood for 10 min, and then the surface water was removed using filter paper. The samples were accurately weighed and marinated with Chinese rice wine (5% based on raw meat weight, *w*/*w*) and a salt solution (the salt addition amount was based on raw meat weight, *w*/*w*) based on experimental design. After marinating, the samples were vacuum packaged and then cooked in a water bath at 70 °C for 10 h (the cooking conditions in the sous vide treatment were selected according to our preliminary experiments). The internal temperature was monitored by a digital thermometer (TES-1384, Tes Electrical Electronic Co., Taibei, China) equipped with a thermocouple and reached 68.5 °C after heating for 2 h. After heating, the samples were chilled immediately in cold water (4 °C) for 0.5 h.

### 2.3. Experimental Design

The brining condition ranges for duck meat were chosen based on the reported literature. For example, duck breasts were marinated in salt solutions of 5%, 15%, and 25% at 4 °C for 72 h [18], Pekin ducks were dry-cured with salt contents of 4%, 6%, and 8% at 4 °C for 3 h [31], duck breasts were dry-salted (10%) for 3 h and pickled in a brine solution for 2 h at room temperature [32], geese were dry-cured with salt contents of 4% and 8% at 4 °C for 24 h [12], and the sliced pork was cured in salt solution from 0% to 10% for 0 to 10 h [16]. During our preliminary experiment, duck legs were marinated with a 6% salt solution at 4 °C for 12 h, and the results showed that the samples had better sensory quality. Therefore, for single-factor analysis, salt concentrations varied from 0% to 15% (0%, 3%, 6%, 9%, 12%, 15% in each group), brining time varied from 4 to 12 h (4 h, 6 h, 8 h,10 h, 12 h in each group), and brining temperature varied from 4 to 20 °C (4 °C, 8 °C, 12 °C, 16 °C, 20 °C). A total of 16 treatments (*n* = 12) were analyzed in single-factor analysis. Response surface methodology (RSM) was utilized to evaluate the independent variables (brining time, X_1_; brining temperature, X_2_; salt concentration, X_3_) on the sensory overall mean score (Y) of the sous vide cooked duck meat. The Box–Behnken design with three levels of each variable was carried out to evaluate the influence of three independent variables on sensory overall mean score. A 3 factor-3 level test was designed to further optimize the brining conditions of the sous vide cooked duck meat. The project included 17 trials, and variance analysis (ANOVA) of the test results was performed via the Design Expert V8.0.6 software to determine the regression coefficient of X_1_, X_2_, X_3_, X1^2^, X_2_^2^, X_3_^2^, X_1_X_2_, X_1_X_3_, and X_2_X_3_ [7]. The analyses of each index were conducted in triplicate.

### 2.4. Sensory Evaluation

Different pickled sous vide cooked duck meat (with skin) was assessed by a well-trained panel consisting of 15 people aged 20–30 (8 females and 7 males) who had performed studies related to food products using the method of quantitative descriptive analysis (QDA). Sensory analysis took place in a sensory laboratory subjected to international standards [33]. Before the sensory descriptive analysis, the members were trained in four preliminary sessions for 4 weeks, and each session was held for approximately 2 h to become familiar with duck meat. During the training sessions, the experts defined and developed each sensory characteristic of the samples until they showed consistent results. The scores of each sensory attribute were represented as a 10-point scale. Sensory attributes of color (surface color of meat; 10 = intense dark red, 1 = bright color), texture (the integrity and structural tightness of the samples; 10 = elastic and dense muscle tissue, 1 = loose, unshaped muscle tissue), meaty (50 g of duck meat was boiled in 100 mL of water for 1 h; 10 = high intensity, 1 = low intensity), fatty (50 g of fat from duck meat was boiled in 100 mL of water for 1 h; 10 = high intensity, 1 = low intensity), and salty appropriateness (the appropriateness of saltiness; 10 = liked very much, 1 = disliked very much) were used to analyze the quality of duck meat samples brining with the salt solution. Moreover, the overall mean score (the sum of 20% of the weight of the above-mentioned five indicators) was also used to evaluate the samples.

Prior to sensory evaluation, the samples were heated in a water bath at 70 °C for 10 min. The cooked whole duck legs and the cut duck meat (with skin) were served in transparent plastic containers with covered lids. The evaluation was conducted in a separate sensory evaluation chamber where the samples were randomly assigned to group members, and unsalted crackers and water were provided as a palate cleanser between samples. The sensory analysis was carried out using a sensory form on which the assessors evaluated and scored each attribute. The sensory quality of each specimen of duck meat was assessed by calculating and plotting an average score for all traits.

### 2.5. Volatile Compounds Analysis

The volatile compounds of different brining duck meat samples (with skin) were identified by gas chromatography coupled with mass spectrometry (GC-MS) after headspace solid-phase microextraction (HS-SPME) according to Zhang et al. [34].

A total of 3 g of the samples with an internal standard (10 µL of 1,2-dichlorobenzene solution, 0.0570 µg/µL in methanol) were placed in a 20 mL vial. During extraction, the samples were immersed in a water bath at 55 °C for 30 min and exposed to a carboxen/polydimethylsiloxane (CAR/PDMS, 75 μm) fiber with an equilibration. After extraction, the solid-phase microextraction (SPME) fiber was transferred to the injector of the GC system for 7 min, which was in the splitless mode at 250 °C. The volatile substances were separated using a capillary DB-5MS column (30 m × 0.25 mm). The oven temperature gradient started at 40 °C for 5 min, moved to 4 °C/min to 200 °C for 2 min, and raised to 250 °C at 20 °C/min for 7 min.

The identification of volatile compounds was carried out by comparing the mass spectra of the compounds with the mass spectra libraries (NIST 14 and WILEY 11 databases) and comparing the retention indexes (RIs) with the published studies. RIs were calculated according to the homogenous series n-alkane (C_7_–C_40_) standard in the same chromatographic conditions. The concentration of the flavor components was calculated by comparing their areas with the internal standard, and the content of the odor-active compounds was calculated by external standards.

### 2.6. Odor Activity Value (OAV)

The OAV was calculated by dividing the content of the compound by its threshold value [28].

### 2.7. Determination of Weight Loss, Color, NaCl Level, TBARS Value, Shear Force, and Textural Properties

#### 2.7.1. Weight Loss

Weight loss was calculated using the following Formula (1), where *mi* was the weight of the duck meat before brining and cooking and *mf* was the weight of duck meat after cooking.

(1) $$\text{Weight loss}\;(\%)=\frac{mi-mf}{mi}\times \;100$$

#### 2.7.2. Color Analysis

The color on the surface of duck meat without skin was detected by a WR-10 colorimeter (Shenzhen Weifu Optoelectronic Technology Co., Ltd., Shenzhen, China) with a D65 light source and a 10° observer. The samples were exposed to the air to bloom for 30 min prior to taking color measurements. After calibration with a white standard board, the values of L* (lightness), a* (redness), and b* (yellowness) in the samples were recorded.

#### 2.7.3. NaCl Level of Duck Meat

The NaCl level of duck meat (with skin) was determined by indirect precipitation titration. The titration method used a silver nitrate standard solution titration, water samples with potassium chromate solution as an indicator, and a silver nitrate standard solution titration of chloride content [35].

#### 2.7.4. TBARS Value Analysis

TBARS value, expressed as mg malondialdehyde (MDA)/kg, was measured using the colorimetric method at 532 nm, according to the method of Chen et al. [36].

#### 2.7.5. Texture and Shear Force Analysis

The texture properties (hardness, cohesiveness, elasticity, adhesiveness, chewiness) of the brined duck meat were determined using a texture analyzer (Ensoul Technology Ltd., Beijing, China) equipped with a cylindrical probe (SMP P/50, 50 mm diameter), according to the method of Zhang et al. [34]. The cooled and deboned duck meat (cut into 1 × 1 × 1 cm^3^ cubes, without skin) was compressed in two cycles. The pre-test, test, and post-test speeds were 2.0 mm/s, 6.0 mm/s, and 5.0 mm/s, respectively. The shape variable was 60%, and the trigger force was 0.5 N. The determination of shear force was referred to the method of Zhu et al. [37] using a digital meat tenderness detection instrument (C-LM3B, Beijing, China). The fascia and epidermis of the duck meat were removed, and then the samples were divided into 3 × 1 × 1 cm sizes along the muscle fiber direction. The shear force value of each sample was recorded.

### 2.8. Free Amino Acid Analysis

The composition and content of free amino acids in salt-brined duck meat (with skin) were analyzed and determined by an automatic amino acid analyzer (Agilent 1100, Agilent Technologies, Santa Clara, CA, USA), according to Zhan et al. [38]. A total of 1000 g of the sample was accurately weighed and mixed with 10 mL of a 5% trichloroacetic acid solution. After ultrasonic extraction for 20 min, the sample was left to stand for 2 h. The solution was filtered and centrifuged (4000 r/min, 20 min).

### 2.9. 5′-Nucleotide Analysis

High-performance liquid chromatography (HPLC) was used to determine the 5′-nucleotide content of duck meat (with skin). The extraction and detection of 5′-nucleotides referred to the method of Pei et al. [39] with slight modifications. A total of 1 g of the sample was accurately weighed (accurate to 0.001 g) and mixed with petroleum ether to extract the oil in the sample, and the operation was repeated 3 times. After treatment, the sample was dissolved in 50 mL of distilled water, extracted with a boiling water bath for 30 min, and filtered through a 0.22 µm water filtration membrane. The determination was performed on a Sunfire C18 column (4.6 mm × 250 mm × 5 µm) at 30 °C with a detection wavelength of 254 nm. The eluents consisted of methanol, distilled water, and KH_2_PO_3_ (5/95/0.05, v:v:v) at a flow rate of 0.6 mL/min. 5′-nucleotides were quantified by external standard method.

### 2.10. Statistic Analysis

All the experiments were conducted in triplicate, and the results were shown as mean ± standard deviation. Data were evaluated by one-way analysis of variance (ANOVA) using SPSS 23.0 (SPSS Inc. Chicago, IL, USA). When the *p*-values were less than 0.05, analyzed by the Tukey test, the model was considered significant. Data from RSM was performed by Design Expert V8.0.6 software (State-Ease Inc., Minneapolis, MN, USA).

## 3. Results and Discussion

### 3.1. The Effects of Salt Concentration on the Sensory Quality, Flavor Components, Physicochemical Indexes, and Texture Properties of Duck Meat

**Figure 1:**
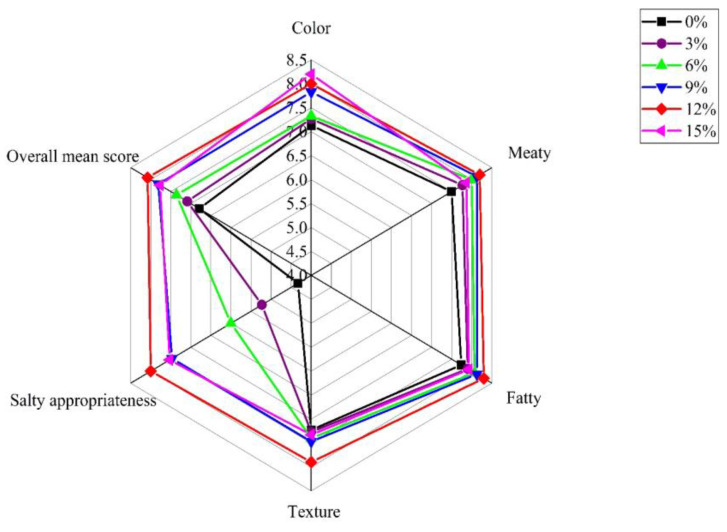
The sensory results of duck meat samples prepared in different salt concentrations.

The salt brining concentration varied from 0% to 15% when considering the influence of salt concentration on the eating quality of sous vide cooked duck meat, and the brining process was carried out at 4 °C for 12 h in single-factor experiments (the initial brining temperature and time were selected based on the published studies. The brining treatment in meat products was usually carried out at 4 °C, and this temperature had been used in Pekin duck [31], geese [12], and chicken breasts [25]. Moreover, Xu et al. reported that sauced duck was marinated in a salt solution of 5% at 4 °C for 12 h, and then dehydrated and marinated with sauce [40]). The sensory evaluation results of different brined duck meat samples are shown in Figure 1. The color, texture, meaty, fatty, salty appropriateness, and overall mean score were used as sensory attributes.

The sensory analysis in Figure 1 demonstrated that the six sensory attributes were significantly affected by salt concentration (*p* < 0.05). The scores of the six sensory attributes in the samples marinated without salt were the lowest, indicating that salt brining treatment could improve the sensory quality of sous vide cooked duck meat. With the increase in salt concentration from 0% to 12%, the scores of meaty, fatty, salty appropriateness, texture, color, and overall mean score in duck meat increased, while an opposite trend was found except the color note when the concentration ranged from 12% to 15%. In general, duck meat brining at a salt concentration of 12% had better sensory quality.

**Figure 2:**
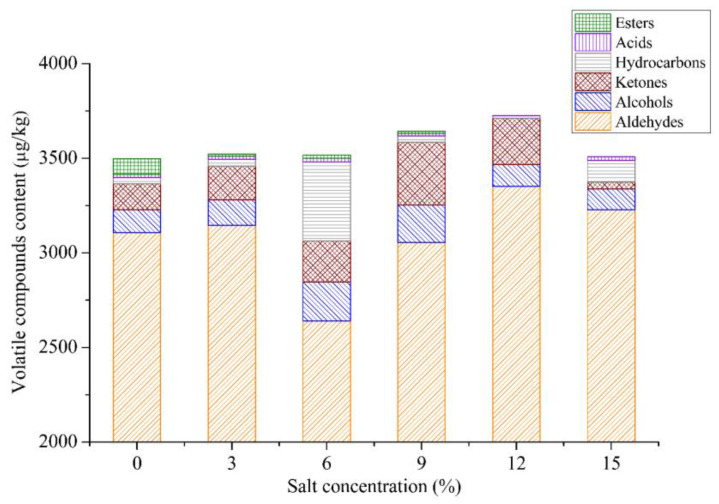
Changes of volatile compound content in duck meat at different salt concentrations.

The effect of different salt concentrations on the types and contents of flavor components of sous vide cooked duck meat by GC-MS is shown in Figure 2. There was a significant difference in the total number of flavor substances among samples brined with different salt concentrations. A total of fifty-eight volatiles were identified in duck meat samples, including eighteen aldehydes, nine alcohols, three ketones, two phenols, nineteen hydrocarbons, one acid, and six esters. Aldehydes contributed more to the overall aroma of duck meat samples due to their high concentration (accounting for 75.07~92.02% of the total volatiles) and low odor thresholds [41]. Aldehydes were primarily formed from the oxidation of unsaturated fatty acids, and the majority of them had meaty and fatty aromas [42]. With the increase in salt concentration, the total number of flavor substances presented an increasing trend at salt concentrations from 0% to 12%, and then decreased from 12% to 15%. The highest contents of flavor compounds and aldehydes were all detected in the samples brined at a salt concentration of 12%. The suitable salt curing concentration promoted the formation of flavor substances. The reason might be that a certain content of sodium chloride could promote the activity of lipase and fat oxidase, and high salt concentration (15%) led to the inhibition of lipoxygenase activity, which could affect the formation of flavor substances [17,43]. The GC-MS results were consistent with the sensory data that the samples brined at a salt concentration of 12% had the highest scores of meaty and fatty notes. Therefore, when the salt concentration was 12%, the samples had the highest total flavor substances and aldehydes, which indicated that the duck meat had better flavor quality combined with the sensory analysis.

The changes in color, NaCl level, weight loss, and the TBARS value of sous vide cooked duck meat brined with different salt concentrations were shown in Table 1. Salt concentration had a significant effect on the color (L*, a*, b*), NaCl content, and the TBARS value of duck meat (*p* < 0.05). Meat color was an indicator affecting the quality of meat. The a* value of duck meat had an increasing trend with the increase in salt concentration. The increase in sodium chloride content could accelerate myoglobin oxidation, causing metmyoglobin formation [25]. The L* values exhibited a decreasing trend with the increase in salt concentration, which might be associated with the decreased lighter scattering due to the increase in water-holding capacity [25]. The increase in a* values in the samples was consistent with the increased score of color note in the sensory analysis. The NaCl level of duck meat ranged from 0.00% to 4.83%, and the highest NaCl level was obtained from the samples brined at a salt concentration of 15%. The higher osmotic pressure in the salt solution could increase the NaCl level of duck meat. The highest NaCl content in the samples brined at 15% imparted a higher saltiness, resulting in a decreased score of salty appropriateness in the sensory analysis. The weight loss decreased along with the increase in salt content. The addition of sodium chloride could increase the water-holding capacity of meat and thus decrease weight loss. The result was consistent with the previous study. Villamonte et al. found that the cooking loss of pork meat batters decreased with the increased salt concentration [44].

The TBARS value was an important attribute in evaluating the degree of lipid oxidation in meat products. The TBARS value reflected the number of secondary oxidation products formed from unsaturated fatty acid degradation and the production of carbonyl compounds, which generated an undesirable aroma in oxidized food [45]. The TBARS value of duck meat significantly decreased with the salt concentration ranging from 0% to 3%. This result was consistent with the reported literature. Sárraga et al. found that the jerky product added with a lower salt content (1–3%) produced lower TBARS values than unsalted samples, while the samples with the salt addition of 5% produced higher TBARS values [46]. The TBARS value of duck meat brined at a salt concentration of 9% was the highest, followed by the samples brined at a salt concentration of 12%. The reason might be that the increase in salt content induced the catalytic effect of NaCl to promote oxidation, which led to an increase in the lipid oxidation activity [47]. Then, the TBARS value went down to a salt concentration of 15%, which was attributed to lipid peroxidation. Lipid peroxidation could lead to the oxidation of sulfhydryl groups, and this would result in the inactivation of lipoxygenase activity [12]. The TBARS results were consistent with GC-MS data that the samples brined at the concentration of 15% had a lower content of flavor substances.

The texture value had a crucial effect on the sensory quality of meat products. Shear force could reflect the meat's tenderness, and a smaller shear force value meant that meat had higher tenderness [37]. The effect of different salt concentrations on the texture properties and shear force of sous vide cooked duck meat is shown in Table 2. The results showed that the hardness and shear force values of duck meat gradually increased with the increase in salt addition. This was consistent with the experimental results of Du et al. [18] and Ozuna et al. [48]. The reason was that the high salt concentration in the brine resulted in great NaCl levels in duck meat. The increase in the NaCl level induced meat muscle shrinks, resulting in hardening in the textural analysis [48]. The elasticity values in samples brined at 15% and the cohesiveness values in duck meat brined at salt concentrations of 9% and 12% were significantly higher than those brined without salt addition (*p* < 0.05). The chewiness of the samples first increased and then decreased with the increase in salt concentration, and the highest value was detected in duck meat brined at a salt concentration of 12%.

The above results indicated that duck meat brined at a salt concentration of 12% had better sensory and flavor quality, a relatively higher L* value and a* value, and moderate tenderness performance. Therefore, a salt concentration of 12% was chosen for the central level of RSM.

### 3.2. The Effects of Brining Time on the Sensory Quality, Flavor Components, Physicochemical Indexes, and Texture Properties of Duck Meat

**Figure 3:**
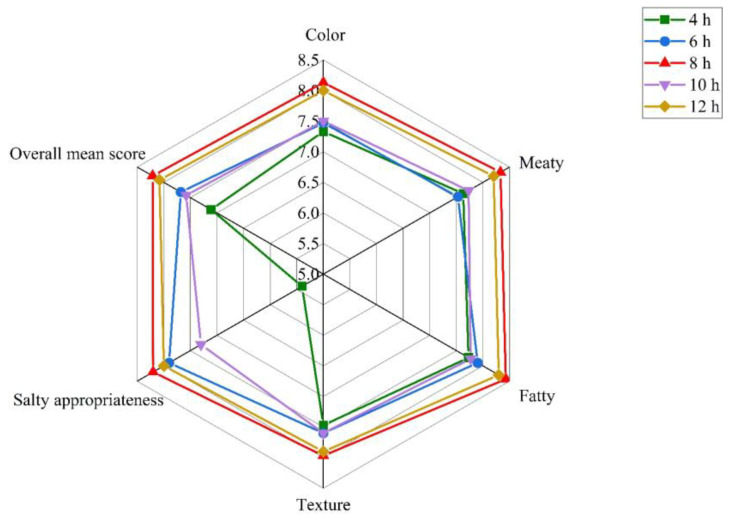
Results of the sensory evaluation of duck meat at different salt brining times.

The effect of brining time ranging from 4 h to 12 h on the eating quality of sous vide cooked duck meat was analyzed, and the brining treatment was conducted with a salt concentration of 12% at 4 °C. The sensory evaluation demonstrated that color, meaty, fatty, salty appropriateness, and overall mean score were significantly affected by brining time (*p* < 0.05). As shown in Figure 3, the scores of the six sensory attributes increased with the increase in brining time from 4 h to 8 h, decreased significantly at a brining time of 10 h, and then increased at 12 h. When the brining time was 4 h, the overall mean score in duck meat was the lowest in all the samples. The scores of the six sensory indexes in duck meat were the highest at a brining time of 8 h, indicating that the samples had better meaty and fatty flavor, a more intact tissue state, and suitable saltiness.

**Figure 4:**
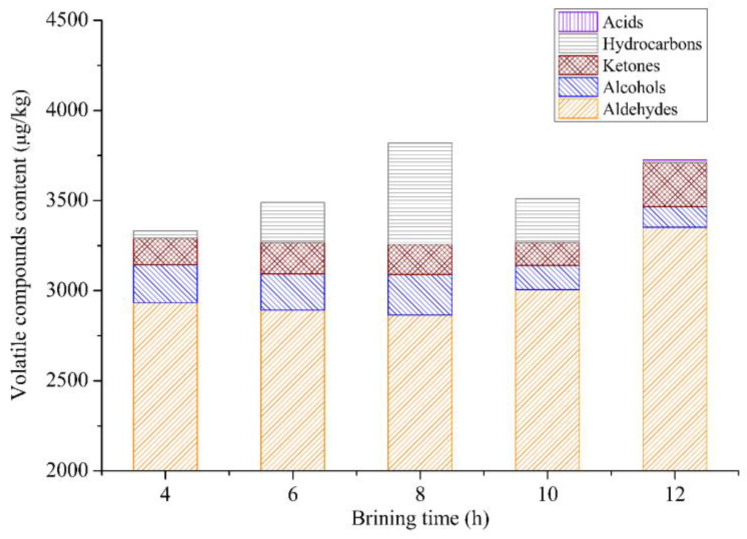
Changes of volatile compound content in duck meat with different brining times.

The flavor substances of sous vide cooked duck meat pretreated with different salt brining times are shown in Figure 4. With an increase in brining time (4–12 h), the total content of the volatile compounds increased, then decreased, and then increased again. The total number of volatiles in samples brined for 8 h or 12 h was significantly higher than in other samples. The total number of flavor substances were the highest in samples brined for 8 h. GC-MS results showed that aldehydes were the most flavor substances in duck meat. The majority of aldehydes had a fatty and meaty odor, which contributed greatly to the overall aroma of duck meat [36]. The content of aldehydes was the highest in the samples at a brining time of 12 h, followed by the duck meat brined for 10 h. Alcohols were mainly derived from lipid oxidation and could partly contribute to the whole meat aroma [49]. 1-octen-3-ol had a mushroom aroma and was the major alcohol in brined duck meat. Among the samples brined with different brining times, the contents of total alcohols and 1-octen-3-ol in duck meat brined for 8 h were higher than those in other groups. Ketones were mainly derived from the Strecker degradation of amino acids or lipid oxidation and often had a creamy and fruity odor [36]. The contents of ketones were relatively higher in the samples treated at a brining time of 12 h. Acids with a pungent odor were only detected in the samples brined for 12 h. GC-MS results indicated that duck meat brining for 8 h or 12 h had better flavor characteristics, which was consistent with the sensory data.

The results of the physicochemical indexes in duck meat treated with different brining times are listed in Table 3. With the extension of brining time, the L* value and weight loss of duck meat gradually decreased, and then the a* value and the NaCl content of duck meat gradually increased. The changes of the L* values and a* values in duck meat coincided with the previous report [50]. The decrease in L* values in the samples was associated with the water-holding capacity caused by the increase in the NaCl content of duck meat [50]. Brining time had a significant effect on NaCl content and the TBARS values of the samples (*p* < 0.05). The NaCl content was the highest in samples brined for 10 h and the lowest in samples brined for 4 h. The results were consistent with the sensory data that higher and lower NaCl content in duck meat had an effect on the saltiness and caused lower values of salty appropriateness. With the increase in brining time, the TBARS values first increased and then decreased. The highest TBARS value was detected in duck meat brined for 6 h (5.87 mg/kg), followed by the samples brined for 10 h. A certain level of sodium chloride could promote lipid oxidation, and with the increase in brining time, duck meat marinated in a high concentration of a salt solution for a long time might inhibit lipid oxidation [51].

According to Table 4, the hardness and the shear force value first increased at the brining time from 4 h to 8 h, decreased at 10 h, and then reached the highest in the samples brined for 12 h. This was consistent with the findings of Wang et al. [52]. The hardness of meat was associated with the denaturation and aggregation of myofibrillar proteins, and the increase in hardness value in the samples brined for 12 h was related to the aggregation of proteins [25,53].

To sum up, the samples brined for 8 h had the highest scores of all the sensory attributes, relatively higher flavor compounds, an L* value and a* value, and moderate tenderness. Thus, a brining time of 8 h was used for the central level of RSM.

### 3.3. The Effects of Brining Temperature on the Sensory Quality, Flavor Components, Physicochemical Indexes, and Texture Properties of Duck Meat

**Figure 5:**
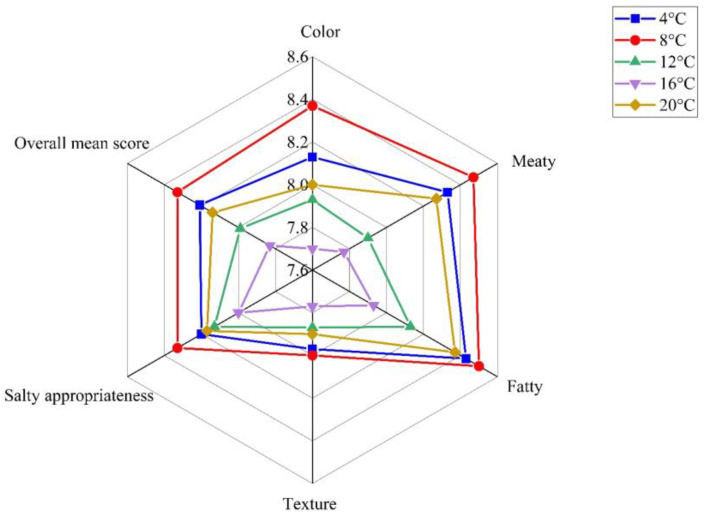
Sensory results of the duck meat at different salt brining temperatures.

The influence of brining temperature ranging from 4 °C to 20 °C on the eating quality of sous vide cooked duck meat was investigated, and the experiment was conducted at a salt concentration of 12% for 8 h. Sensory results of different brined duck meat samples are shown in Figure 5. The results demonstrated that color, meaty, fatty, and overall mean score were significantly affected by brining temperature (*p* < 0.05). The scores of meaty, fatty, color, salt appropriateness, texture, and overall mean score in the samples increased from 4 °C to 8 °C, decreased at a brining temperature from 8 °C to 16 °C, and then increased at 20 °C. When the brining temperature was 8 °C, sous vide cooked duck meat had the highest scores of all the sensory indexes, indicating that an appropriate brining temperature could improve the sensory quality of duck meat.

**Figure 6:**
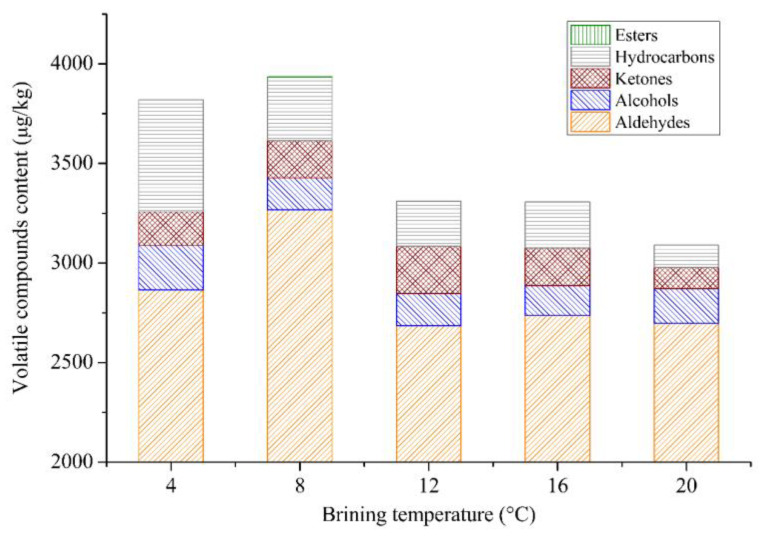
The content of the volatile flavor compounds in duck meat at different brining temperatures.

The effect of salt brining temperature on the flavor compounds of sous vide cooked duck meat detected by GC-MS is shown in Figure 6. The total number of volatiles in the samples brined at 4 °C or 8 °C was significantly higher than in other samples. The results indicated that the total number of volatile compounds first increased and then decreased with the increase in brining temperature. The reason might be that the chemical structure of some volatile substances was unstable, and rising temperatures accelerated their degradation, resulting in lower content [54]. The total content of flavor substances and aldehydes in duck meat brined at 8 °C was higher than in other samples. This result was consistent with the higher scores of meaty and fatty aromas in the sensory analysis.

The physicochemical indexes of duck meat at different brining temperatures are listed in Table 5. The weight loss of duck meat decreases with the increase in brining temperature. The reason might be that the brining temperature had an effect on the penetration rate of the salt solution. The NaCl content in duck meat increased with the increase in brining temperature. Combined with the sensory analysis, when the brining temperature was 8 °C, the samples had a higher value of salty appropriateness compared with other samples. The L* and a* values in the samples brined from 4 °C to 16 °C were higher than those brined at 20 °C. The results were similar to the literature in that the L* and a* values of bacon decreased when the brining temperature ranged from 16 °C to 19 °C [55]. The reason might be that the brining temperature induced some chemical reactions, which affected the color of the meat [55]. When the brining temperature was 8 °C, the samples had medium lightness and redness and the highest score of salty appropriateness.

The texture properties of duck meat at different brining temperatures are listed in Table 6. The brining temperature had no significant effect on hardness but had a significant effect on shear force (*p* < 0.05). When the brining temperature was 8 °C, the hardness of duck meat was relatively higher than in other samples. The shear force value showed a decreasing trend, which might be because with the increase in brining temperature, the integrity of the fascicular membrane and endomyoma was damaged to a greater extent, and the myofibrillar structure changed and gradually degraded.

In conclusion, the samples brined at 8 °C had better sensory and flavor quality and a relatively higher L* value and a* value. Thus, a brining temperature of 8 °C was selected as the central level of RSM.

### 3.4. Optimization of Salt Brining Conditions by RSM

RSM as a statistical tool was frequently applied to optimize a response value that was influenced by one or more independent variables. In our study, brining time (X_1_), brining temperature (X_2_), and salt concentration (X_3_) were selected as independent variables, and sensory overall mean score (Y) was used as the dependent variable. Therefore, a 3-level and 3-factorial design and the sensory overall mean score results detected from different treatments are shown in Table 7. The response surface model equation through a multiple regression analysis was established as follows:

Y = 8.57 + 8.407X_1_ − 0.064X_2_ − 0.068X_3_ − 0.043X_1_X_2_ + 0.018X_1_X_3_ + 0.039X_2_X_3_ − 0.02X_1_^2^ − 0.21X_2_^2^ − 0.35X_3_^2^

The ANOVA result of the response model is shown in Table 8. The F value for the model was 23.65, and the *p*-value was 0.0002, which was less than 0.001. Therefore, the model given by Design Expert was significant [56]. The determination coefficient R^2^ of the sensory overall mean score was 0.9682. These results indicated a good agreement between the experimental and predicted values, and the mathematical model of the sensory overall mean score in our study was reliable.

The results in Table 8 revealed that X_2_ (brining temperature), X_3_ (salt concentration), X_1_^2^, X_2_^2^, and X_3_^2^ had significant effects on the sensory overall mean score of duck meat (*p* < 0.05). The optimum salt brining conditions of sous vide cooked duck meat obtained by Design Expert were shown as follows: a brining time of 8.03 h, a brining temperature of 7.35 °C, and a salt concentration of 11.69%. The predicted maximum value of the overall mean score was found to be 8.58. Under the optimum salt brining conditions, the actual overall mean score of sous vide cooked duck meat was 8.59, which proved that the experimental data were close to the theoretical predicted value. Thus, the model could be used to optimize the salt brining conditions of duck meat.

### 3.5. A Comparison of the Quality of Unsalted and Salt-Optimized Brined Duck Meat Cooked with the Sous Vide Method

#### 3.5.1. Analysis of Physiochemical Indexes

The indexes of salted duck meat were determined according to the optimized technological parameters, which could reflect the palatability and acceptability of the finished product. The measured texture properties of duck meat were shown as follows: a hardness of 35.37 N, a cohesiveness ratio of 0.32, an elasticity of 2.39 mm, an adhesiveness of 11.93 N, a chewiness of 13.40 mJ, and a shear force of 36.21 N. Moreover, the L* value was 50.21, the a* value was 5.25, the b* value was 11.30, the NaCl content was 3.21%, the weight loss was 34.49%, and the TBARS value was 4.06 mg/kg. Compared with the unsalted products, the brined samples under these optimum conditions had higher levels of hardness, a* values, TBARS values, and moderate NaCl content, and lower levels of weight loss and L* values. The results showed that the brined duck meat products had better color and moderate NaCl content.

#### 3.5.2. Analysis of Flavor Components and the Odorants

The difference in flavor components and the odorants between the duck meat without the salt brining process and with the optimum salt brining conditions was analyzed by GC-MS combined with OAV values (Table 9). In total, 39 flavor substances were detected in the unsalted or the optimum salted duck meat, but the types and content of the flavor substances in the two treatment samples were different. There were twenty aldehydes, two alcohols, three ketones, ten hydrocarbons, two esters, and two acids in samples without salt brining treatment, while there were eighteen aldehydes, six alcohols, one ketone, thirteen hydrocarbons, and one ester in samples with the optimum salt brining treatment. Moreover, the total flavor content of the brined samples increased by 7.60% compared with the unsalted samples.

Flavor perception and the contribution to the overall aroma of meat for each component were not only associated with the concentration of flavor substances but also referred to the odor threshold of flavor compounds. Thus, the OAV values of volatile compounds were calculated and listed in Table 9. The components with an OAV greater than 1 were the key odor-active compounds that contributed to the overall flavor [51]. As shown in Table 9, twelve aldehydes (such as pentanal, hexanal, heptanal, and so on), two alcohols (1-heptanol, 1-octen-3-ol), and two ketones (1-octen-3-one, 2-methyl-3-octanone) had high OAV values (OAV > 1). The results indicated that there were 12 and 15 odorants in unsalted and the optimum salted samples, respectively. In unsalted duck meat, 2,4-decadienal had the highest OAV (640.4), followed by hexanal (522.39), 1-octen-3-ol (432.8), and cis-4-decenal (307.5) (OAV > 100). For the salted duck meat samples, cis-4-decenal had the highest OAV value (1820), followed by decanal (779.43), 2,4-decadienal (670), (E,E)-2,4-decadienal (532.22), hexanal (527.74), 1-octen-3-ol (224.17), octanal (136.31), and (E)-2-nonenal (124.58) (OAV > 100). The OAVs of ten odorants (pentanal, heptanal, octanal, (E)-2-nonenal, cis-4-decenal, decanal, 2,4-decadienal, (E,E)-2,4-decadienal, 1-heptanol, 2-methyl-3-octanone) were significantly higher, while the OAVs of six odorants (hexanal, (E)-2-octenal, nonanal, (E,E)-2,4-nonadienal, 1-octen-3-ol, 1-octen-3-one) were relatively lower in the optimum salted duck meat compared to the unsalted samples.

#### 3.5.3. Analysis of Taste Components

There were seventeen kinds of free amino acids in sous vide cooked duck meat (Table 10), which could be divided into four kinds according to their flavor characteristics and taste, namely umami, sweet, tasteless, and bitter. There were no significant differences in total free amino acid content between the two groups, and the total free amino acid content of the SV unsalted samples (241.8 ± 4.33 mg/100 g) was higher than in the SV the optimum salted samples (227.16 ± 5.70 mg/100 g). The content of sweet amino acids in the SV unsalted samples was mostly higher than the SV salted samples, and only the sweet amino acid of proline was slightly lower. Moreover, the concentration of umami amino acids was higher in the SV unsalted samples. However, the content of bitter amino acids increased after the salt-curing treatment. This might be because the presence of NaCl accelerated the coagulation of amino acids and formed precipitation [60].

The total content of 5′-nucleotides in salt-brined duck meat reached 1771.68 mg/g, 1.10 times that in the unsalted samples. 5′-inosine monophosphate (IMP) was an important indicator of umami taste and an important source of nitrogenous compounds in cooked meat [61]. The amount of IMP in the salt-brined samples was significantly higher than in the unsalted samples, which was consistent with the result of Zhang et al. [62]. 5′-CMP, 5′-UPM, and 5′-GMP were also higher in salt-brined duck meat compared to the uncured samples.

Analysis of free amino acids and 5′-nucleotides indicated that the salt brining treatment had an effect on the content of the taste components.

## 4. Conclusions

The salt brining conditions (salt concentration, brining time, brining temperature) of sous vide cooked duck meat were optimized using response surface methodology. Sensory evaluation indicated that salt curing treatment could improve the scores of fatty, meaty, color, texture, salty appropriateness, and the overall mean score of the samples compared with those without salt addition. The optimum salt curing process conditions were as follows: a brining time of 8.03 h, a brining temperature of 7.35 °C, and a salt concentration of 11.69%. On this basis, duck meat had a better sensory overall mean score (8.59). GC-MS results combined with OAV values showed that the characteristic flavor substances (such as pentanal, heptanal, octanal, (E)-2-nonenal, cis-4-decenal, decanal, 2,4-decadienal, (E,E)-2,4-decadienal, 1-heptanol, 2-methyl-3-octanone) in duck meat were significantly increased by brining treatment, while the minority odorants (hexanal, (E)-2-octenal, nonanal, (E,E)-2,4-nonadienal, 1-octen-3-ol, 1-octen-3-one) decreased slightly. There was no significant difference in free amino acid content between unsalted and the optimum salted duck meat, but the content of 5′-nucleotides in salt-brined duck meat significantly increased, indicating that salting could improve the taste of duck meat.

## Figures and Tables

**Table 1:** The effect of salt concentration on the physicochemical indexes of duck meat.

Indexes	0%	3%	6%	9%	12%	15%
L*	61.96 ± 0.51 ^a^	54.38 ± 1.03 ^b^	51.25 ± 0.06 ^c^	52.26 ± 1.70 ^c^	56.44 ± 2.65 ^b^	42.23 ± 0.46 ^d^
a*	4.81 ± 0.35 ^d^	5.18 ± 0.43 ^cd^	5.46 ± 0.02 ^c^	6.31 ± 086 ^b^	6.12 ± 0.16 ^b^	7.28 ± 0.02 ^a^
b*	10.27 ± 0.04 ^bc^	12.48 ± 0.08 ^a^	12.78 ± 0.09 ^a^	11.03 ± 0.64 ^b^	9.66 ± 2.89 ^c^	11.91 ± 0.02 ^b^
NaCl content/%	0.00 ± 0.00 ^e^	1.14 ± 0.01 ^d^	2.32 ± 0.01 ^c^	2.46 ± 0.01 ^c^	3.79 ± 0.01 ^b^	4.83 ± 0.01 ^a^
Weight loss/%	39.34 ± 0.01 ^a^	36.58 ± 0.01 ^b^	35.67 ± 0.01 ^b^	34.54 ± 0.01 ^c^	32.19 ± 0.01 ^d^	29.76 ± 0.01 ^e^
TBARS (mg/kg)	2.64 ± 0.04 ^c^	1.66 ± 0.02 ^f^	2.21 ± 0.01 ^d^	3.60 ± 0.01 ^a^	3.25 ± 0.01 ^b^	1.77 ± 0.01 ^e^

Letters (a–f) in the superscript of the numbers in each line indicated significant differences (*p* < 0.05).

**Table 2:** The effect of salt concentration on the texture properties of duck meat.

Groups	Hardness /N	Cohesiveness /Ratio	Elasticity /mm	Adhesiveness /N	Chewiness /mJ	Shear Force /N
0%	24.37 ± 2.81 ^c^	0.27 ± 0.02 ^b^	2.08 ± 0.14 ^b^	8.30 ± 3.15 ^b^	20.39 ± 1.73 ^d^	21.78 ± 0.67 ^e^
3%	28.10 ± 3.07 ^b^	0.32 ± 0.02 ^ab^	2.39 ± 0.45 ^ab^	9.07 ± 1.42 ^b^	21.94 ± 2.26 ^cd^	26.11 ± 2.10 ^d^
6%	29.63 ± 2.36 ^b^	0.32 ± 0.02 ^ab^	2.40 ± 0.12 ^ab^	11.90 ± 3.68 ^b^	25.42 ± 0.89 ^bc^	28.95 ± 3.28 ^d^
9%	31.77 ± 1.12 ^ab^	0.33 ± 0.05 ^a^	2.45 ± 0.34 ^ab^	16.70 ± 0.40 ^a^	26.49 ± 2.88 ^b^	36.88 ± 1.41 ^c^
12%	34.40 ± 0.53 ^a^	0.33 ± 0.02 ^a^	2.40 ± 0.12 ^ab^	12.47 ± 1.43 ^b^	29.87 ± 4.11 ^a^	42.61 ± 0.23 ^b^
15%	35.17 ± 1.16 ^a^	0.32 ± 0.03 ^ab^	2.82 ± 0.70 ^a^	10.07 ± 1.86 ^b^	26.21 ± 2.79 ^bc^	48.28 ± 0.35 ^a^

Letters (a–e) in the superscript of the numbers in each column indicated significant differences in data (*p* < 0.05).

**Table 3:** The effect of salt brining time on the physicochemical indexes of duck meat.

Indexes	4 h	6 h	8 h	10 h	12 h
L*	67.92 ± 0.07 ^a^	60.76 ± 1.15 ^b^	60.27 ± 0.43 ^b^	57.34 ± 0.14 ^c^	56.44 ±2.65 ^c^
a*	4.74 ± 0.01 ^c^	4.76 ± 0.09 ^c^	5.66 ± 0.08 ^b^	5.67 ± 0.03 ^b^	6.12 ±0.16 ^a^
b*	12.14 ± 0.02 ^c^	17.12 ± 1.63 ^a^	13.10 ± 0.08 ^bc^	13.06 ± 0.05 ^bc^	9.66 ± 2.89 ^c^
NaCl content/%	2.08 ± 0.01 ^c^	2.93 ± 0.01 ^b^	3.79 ± 0.01 ^a^	3.98 ± 0.01 ^a^	3.79 ± 0.01 ^a^
Weight loss/%	35.58 ± 0.01 ^a^	34.30 ± 0.01 ^a^	32.68 ± 0.01 ^b^	32.81 ± 0.01 ^b^	32.19 ± 0.01 ^b^
TBARS (mg/kg)	3.96 ± 0.01 ^d^	5.87 ± 0.01 ^a^	4.15 ± 0.05 ^c^	4.53 ± 0.02 ^b^	3.25 ± 0.02 ^e^

Letters (a–e) in the superscript of the numbers in each line indicated significant differences (*p* < 0.05).

**Table 4:** The effect of salt brining time on the texture properties of duck meat.

Groups	Hardness /N	Cohesiveness/Ratio	Elasticity /mm	Adhesiveness /N	Chewiness /mJ	Shear Force/N
4 h	30.12 ± 2.05 ^b^	0.36 ± 0.04 ^a^	3.33 ± 0.42 ^a^	8.93 ± 1.62 ^c^	44.37 ± 11.65 ^a^	29.50 ± 2.13 ^c^
6 h	30.12 ± 2.05 ^b^	0.36 ± 0.04 ^a^	3.33 ± 0.30 ^a^	8.93 ± 1.44 ^c^	44.37 ± 0.89 ^a^	29.05 ± 0.98 ^c^
8 h	33.23 ± 1.78 ^a^	0.30 ± 0.05 ^c^	3.43 ± 0.28 ^a^	9.97 ± 0.32 ^b^	24.01 ± 4.70 ^d^	37.21 ± 2.14 ^b^
10 h	32.16 ± 0.87 ^b^	0.34 ± 0.04 ^b^	3.36 ± 1.17 ^a^	9.28 ± 0.46 ^c^	37.58 ± 9.39 ^b^	34.21 ± 0.18 ^b^
12 h	34.40 ± 0.53 ^a^	0.33 ± 0.02 ^b^	2.40 ± 0.12 ^b^	12.47 ± 1.43 ^a^	29.87 ± 4.11 ^c^	42.61 ± 0.23 ^a^

Letters (a–d) in the superscript of the numbers in each column indicated significant differences in data (*p* < 0.05).

**Table 5:** The effect of brining temperatures on the physicochemical indexes of duck meat.

Indexes	4 °C	8 °C	12 °C	16 °C	20 °C
L*	60.27 ± 0.43 ^a^	50.37 ± 1.07 ^b^	51.97 ± 0.96 ^b^	50.93 ± 1.90 ^b^	45.80 ± 1.00 ^c^
a*	5.66 ± 0.08 ^a^	5.20 ± 0.07 ^a^	5.32 ± 0.82 ^a^	5.70 ± 0.31 ^a^	4.92 ± 0.15 ^b^
b*	13.10 ± 0.08 ^a^	11.27 ± 0.18 ^b^	11.26 ± 1.66 ^b^	10.52 ± 0.70 ^c^	11.32 ± 1.03 ^b^
NaCl content/%	3.79 ± 0.01 ^d^	3.88 ± 0.01 ^c^	4.07 ± 0.01 ^c^	4.45 ± 0.01 ^b^	5.11 ± 0.01 ^a^
Weight loss/%	32.68 ± 0.01 ^a^	33.07 ± 0.01 ^a^	33.03 ± 0.01 ^a^	31.67 ± 0.01 ^b^	29.52 ± 0.01 ^c^
TBARS (mg/kg)	4.15 ± 0.05 ^c^	4.03 ± 0.01 ^d^	4.21 ± 0.01 ^b^	4.15 ± 0.01 ^c^	4.40 ± 0.01 ^a^

Letters (a–d) in the superscript of the numbers in each line represented significant differences (*p* < 0.05).

**Table 6:** The effect of brining temperatures on the texture properties of duck meat.

Groups	Hardness /N	Cohesiveness /Ratio	Elasticity /mm	Adhesiveness /N	Chewiness /mJ	Shear Force/N
4 °C	33.23 ± 1.78 ^a^	0.30 ± 0.05 ^ab^	3.43 ± 0.28 ^a^	9.97 ± 0.32 ^b^	24.01 ± 4.70 ^b^	37.21 ± 2.14 ^a^
8 °C	35.95 ± 0.15 ^a^	0.36 ± 0.03 ^a^	2.90 ± 0.24 ^b^	12.82 ± 0.98 ^a^	11.70 ± 0.26 ^c^	35.54 ± 0.66 ^a^
12 °C	35.13 ± 2.59 ^a^	0.32 ± 0.01 ^ab^	2.86 ± 0.23 ^b^	11.17 ± 0.86 ^b^	31.95 ± 2.71 ^a^	36.92 ± 0.20 ^a^
16 °C	35.47 ± 9.42 ^a^	0.29 ± 0.01 ^b^	2.93 ± 0.35 ^b^	10.27 ± 2.99 ^b^	30.74 ± 12.64 ^a^	29.30 ± 1.47 ^b^
20 °C	29.97 ± 5.76 ^a^	0.26 ± 0.06 ^b^	2.52 ± 1.31 ^c^	6.88 ± 3.73 ^c^	11.26 ± 2.33 ^c^	28.51 ± 1.30 ^b^

Letters (a–c) in the superscript of the numbers in each column indicate significant differences in data (*p* < 0.05).

**Table 7:** Box–Behnken design and the corresponding sensory overall mean score results.

Trial	Independent Variables			Overall Mean Score
	Brining Time (h)	Brining Temperature (°C)	Salt Concentration (%)	
1	8	8	12	8.59
2	8	8	12	8.62
3	6	8	9	8.07
4	8	8	12	8.54
5	10	12	12	7.96
6	6	8	15	7.85
7	8	12	15	7.98
8	8	4	9	8.11
9	10	8	15	7.96
10	10	4	12	8.25
11	10	8	9	8.12
12	8	8	12	8.51
13	6	4	12	8.23
14	8	4	15	7.96
15	8	8	12	8.60
16	6	12	12	8.12
17	8	12	9	7.98

**Table 8:** ANOVA for the response surface model.

Sources of Variation	Sum of Squares	Degree of Freedom	Mean Square	F-Value	*p*-Value
Model	1.10	9	0.12	23.65	0.0002
X_1_	5.768	1	5.768	1.148	0.9735
X_2_	0.033	1	0.033	6.35	0.0398
X_3_	0.037	1	0.03	7.09	0.0323
X_1_X_2_	7.261	1	7.261	1.44	0.2685
X_1_X_3_	1.329	1	1.329	0.26	0.6230
X_2_X_3_	6.057	1	6.057	1.20	0.3086
X_1_^2^	0.20	1	0.20	38.98	0.0004
X_2_^2^	0.19	1	0.19	36.70	0.0005
X_3_^2^	0.53	1	0.53	101.72	<0.0001
Residual	0.036	7	5.026		
Lack of fit	0.028	3	9.015	4.44	0.0919
Pure Error	8.126	4	2.031		
Total	1.14	16			

**Table 9:** The effect of the salt curing treatment on the flavor compounds and the odorants of sous vide cooked duck meat.

Code	Compounds	RI ^1^	RI ^2^	Threshold [57] (μg/kg of Water)	Concentration (μg/kg)		OAV ^3^	
					Unsalted	The Optimum Salted	Unsalted	The Optimum Salted
	**Aldehydes**							
AL1	Pentanal	699	698	12	20.17 ± 0.77	262.20 ± 5.96	1.68	21.85
AL2	Hexanal	802	798	4.5	2485.74 ± 196.85	2374.83 ± 48.60	552.39	527.74
AL3	Heptanal	904	905	2.8	2.57 ± 1.11	11.62 ± 0.35	<1	4.15
AL4	Benzaldehyde	958	963	24 [58]	0.06 ± 0.00	9.76 ± 0.58	<1	<1
AL5	(Z)-2-Heptenal	960	963	/	17.09 ± 0.11	10.77 ± 0.53	/	/
AL6	Octanal	1005	1000	0.7	27.48 ± 5.88	95.42 ± 2.41	39.26	136.31
AL7	(E)-2-Octenal	1061	1070	3	15.97 ± 0.88	14.84 ± 0.09	5.32	4.95
AL8	Nonanal	1107	1102	1	81.94 ± 0.56	54.83 ± 0.26	81.94	54.83
AL9	(E)-2-Nonenal	1162	1159	0.19	3.59 ± 0.50	23.67 ± 0.18	18.89	124.58
AL10	cis-4-Decenal	1195	1193	0.004	1.23 ± 0.14	7.28 ± 0.20	307.5	1820
AL11	Decanal	1208	1214	0.3	2.60 ± 0.30	233.83 ± 5.08	8.67	779.43
AL12	2,4-Dimethyl-benzaldehyde	1212	1180	/	0.29 ± 0.06	/	/	/
AL13	(E,E)-2,4-Nonadienal	1218	1215	0.05	1.00 ± 0.26	0.75 ± 0.01	20	15
AL14	(Z)-2-Decanal	1264	1250	/	3.21 ± 0.04	2.30 ± 0.04	/	/
AL15	2,4-Decadienal	1296	1284	0.3	192.12 ± 16.64	201.00 ± 2.50	640.4	670
AL16	Undecanal	1309	1303	12.5	/	/	/	/
AL17	(E,E)-2,4-Decadienal	1320	1345	0.027	/	14.37 ± 0.66	/	532.22
AL18	2-Undecanal	1365	1350	/	1.76 ± 0.13	2.98 ± 0.15	/	/
AL19	Dodecanal	1413	1412	55	0.36 ± 0.02	1.00 ± 0.05	<1	<1
AL20	Tridecanal	1513	1510	70	0.70 ± 0.01	0.50 ± 0.05	<1	<1
AL21	Tetradecaldehyde	1613	1592	/	0.59 ± 0.01	/	/	/
AL22	Pentadecanal	1713	1691	430	0.93 ± 0.04	/	<1	/
	Subtotal				2830.17	3322.96		
	**Alcohols**							
A1	1-Hexenol	876	889	800	/	7.51 ± 0.83	/	<1
A2	1-Heptanol	976	971	5.4 [59]	/	13.54 ± 0.95	/	2.51
A3	1-Octen-3-ol	984	979	1	432.48 ± 126.88	224.17 ± 2.58	432.48	224.17
A4	4-Ethylcyclohexanol	1040	1003	/	/	3.21 ± 0.50	/	/
A5	(E)-2-Octen-1-ol	1072	1069	100	0.28 ± 0.05	10.87 ± 0.31	<1	<1
A6	1-Octanol	1076	1070	190	/	11.17 ± 0.76	/	<1
	Subtotal				432.76	270.47		
	**Ketones**							
K1	Cyclohexanone	894	871	280	50.60 ± 1.70	/	<1	/
K2	2,5-Octanedione	983	971	/	220.47 ± 9.62	/	/	/
K3	1-Octen-3-one	980	978	0.05	2.36 ± 0.27	/	47.2	/
K4	2-Methyl-3-octanone	988	985	50.2	/	108.00 ± 3.07	/	2.15
	Subtotal				273.39	108.00		
	**Hydrocarbons**							
H1	Toluene	756	759	1550 [59]	4.34 ± 0.45	83.85 ± 3.13	<1	<1
H2	Ethylbenzene	855	864	2205 [59]	0.73 ± 0.06	/	<1	/
H3	Decane	1000	1000	10,000	/	2.51 ± 0.86	/	<1
H4	p-Xylene	866	875	490 [59]	0.30 ± 0.07	/	<1	/
H5	1,3-Dimethylbenzene	888	885	1000	1.71 ± 0.13	/	<1	/
H6	Undecane	1100	1100	10,000	2.87 ± 0.08	16.59 ± 1.48	<1	<1
H7	3-Methyl-undecane	1170	1169	/	/	0.99 ± 0.04	/	/
H8	Dodecane	1200	1200	2040 [59]	3.75 ± 0.23	20.18 ± 0.58	<1	<1
H9	Tridecane	1300	1300	2140 [59]	/	0.78 ± 0.26	/	<1
H10	3-Methyl-tridecane	1369	1369	/	0.40 ± 0.02	2.04 ± 0.25	/	/
H11	3-Methylenetridecane	1386	1407	/	/	/	/	/
H12	Tetradecane	1400	1400	1000	1.50 ± 0.02	2.10 ± 0.20	<1	<1
H13	7-Tetradecane	1402	1411	/	0.76 ± 0.13	/	/	/
H14	Cyclodecane	1456	1406	290	/	0.40 ± 0.03	/	<1
H15	5-Methyltetradecane	1454	1456	/	/	0.25 ± 0.05	/	/
H16	Pentadecane	1500	1500	/	0.66 ± 0.12	0.46 ± 0.08	/	/
H17	3-Methylpentadecane	1574	1570	/	/	0.42 ± 0.02	/	/
H18	Hexadecane	1600	1600	/	/	0.40 ± 0.05	/	/
H19	(1-Pentylheptyl)-benzene	1726	1719	/	/	/	/	/
	Subtotal				17.02	130.98		
	**Esters**							
E1	Methyl palmitate	1927	1927	2000	/	0.10 ± 0.01	/	<1
E2	Methyl oleate	2086	2066	/	0.94 ± 0.05	/	/	/
E3	Methyl stearate	2128	2093	/	0.37 ± 0.01	/	/	/
	Subtotal				1.31	0.10		
	**Acids**							
AC1	Acetic acid	689	637	22,000	3.07 ± 0.18	/	<1	/
AC2	Hexadecanoic acid	1961	1977	10,000	3.87 ± 0.02	/	<1	/
	Subtotal				6.94	0.00		
	Total				3561.59	3832.50		

^1^ RI: retention indexes calculated by n-alkanes. ^2^ RI: RI from the database (https://webbook.nist.gov/chemistry/cas-ser/ (accessed on 10 May 2023)). ^3^ OAV: odor activity value.

**Table 10:** The effect of the salt brining treatment on the content of taste compounds in sous vide cooked duck meat.

Taste Component	Taste Characteristics	Unsalted	The Optimum Salted
**Free amino acid**		**Concentration (mg/100 g)**	
Aspartic acid	umami	12.97 ± 0.10	12.85 ± 1.00
Glutamic acid	umami	29.38 ± 1.51	28.58 ± 0.60
Serine	sweet	13.50 ± 0.59	12.20 ± 0.79
Threonine	sweet	14.80 ± 0.54	12.47 ± 0.52
Proline	sweet	6.98 ± 0.87	7.45 ± 0.40
Alanine	sweet	86.95 ± 0.54	74.87 ± 3.61
Glycine	sweet	14.00 ± 0.84	11.90 ± 0.18
Histidine	bitter	4.87 ± 0.16	5.36 ± 0.47
Arginine	bitter	11.17 ± 0.68	13.08 ± 0.73
Tyrosine	bitter	7.82 ± 0.32	6.50 ± 0.38
Valine	bitter	10.56 ± 0.42	10.89 ± 0.51
Methionine	bitter	3.80 ± 0.27	4.43 ± 0.40
Phenylalanine	bitter	4.45 ± 0.40	4.74 ± 0.18
Isoleucine	bitter	3.46 ± 0.50	4.39 ± 0.33
Leucine	bitter	7.35 ± 0.48	8.40 ± 0.34
Lysine	bitter	8.59 ± 0.27	8.23 ± 0.12
Cysteine	tasteless	1.15 ± 0.17	0.81 ± 0.04
Total		241.80 ± 4.33	227.16 ± 5.70
**5′-Nucleotides**		**Concentration (mg/kg)**	
5′-CMP		193.71 ± 12.17	223.66 ± 14.71
5′-AMP		127.56 ± 8.36	104.57 ± 2.43
5′-UPM		894.94 ± 9.83	981.51 ± 10.04
5′-GMP		42.77 ± 4.58	52.89 ± 5.33
5′-IMP		350.94 ± 5.10	409.06 ± 7.01
Total		1609.91 ± 24.93	1771.68 ± 24.35

## Data Availability

The datasets generated for this study are available on request to the corresponding author.
